# Where internal medical patients receive intensive interventions: results from a tertiary-care hospital in Israel

**DOI:** 10.1186/s13584-023-00570-z

**Published:** 2023-05-24

**Authors:** Gideon Leibner, Shuli Brammli-Greenberg, David Katz, Yaakov Esayag, Nechama Kaufman, Adam J. Rose

**Affiliations:** 1 Hebrew University of Jerusalem Braun School of Public Health and Community Medicine, Jerusalem, Israel; 2 Department of Internal Medicine, Shaare Zedek Medical Center and Faculty of Medicine, Hebrew University of Jerusalem, Jerusalem, Israel; 3 Department of Quality and Patient Safety, Shaare Zedek Medical Center, Jerusalem, Israel; 4 Department of Emergency Medicine, Shaare Zedek Medical Center, Jerusalem, Israel

**Keywords:** Intensive care treatments, Intermediate care unit, Intensive care unit, Intensive care supply, Intensive care unit shortage

## Abstract

**Background:**

Patients admitted to internal medicine may be moved to more advanced-care settings when their condition deteriorates. In these advanced care settings, there may be higher levels of monitoring and greater ability to deliver Intensive Medical Treatments (IMTs). To the best of our knowledge, no previous study has examined the proportion of patients at different levels of care who receive different types of IMTs.

**Methods:**

In this retrospective observational cohort study, we examined data from 56,002 internal medicine hospitalizations at Shaare Zedek Medical Center, between 01.01.2016 and 31.12.2019. Patients were divided according to where they received care: general-ward, Intermediate-Care Unit, Intensive Care Unit (ICU), or both (Intermediate-Care and ICU). We examined the rates at which these different groups of patients received one or more of the following IMTs: mechanical ventilation, daytime bi-level positive airway pressure (BiPAP), or vasopressor therapy.

**Results:**

Most IMTs were delivered in a general-ward setting – ranging from 45.9% of IMT-treated hospitalizations involving combined mechanical ventilation and vasopressor therapy to as high as 87.4% of IMT-treated hospitalizations involving daytime BiPAP. Compared to ICU patients, Intermediate-Care Unit patients were older (mean age 75.1 vs 69.1, *p* < 0.001 for this and all other comparisons presented here), had longer hospitalizations (21.3 vs 14.5 days), and were more likely to die in-hospital (22% vs 12%). They were also more likely to receive most of the IMTs compared to ICU patients. For example, 9.7% of Intermediate-Care Unit patients received vasopressors, compared to 5.5% of ICU patients.

**Conclusion:**

In this study, most of the patients who received IMTs actually received them in a general-bed and not in a dedicated unit. These results imply that IMTs are predominantly delivered in unmonitored settings, and suggest an opportunity to re-examine where and how IMTs are given. In terms of health policy, these findings suggest a need to further examine the setting and patterns of intensive interventions, as well as a need to increase the number of beds dedicated to delivering intensive interventions.

## Background

An Intensive Care Unit (ICU) is a unit designed to treat patients with the most difficult conditions, those who need close monitoring or intensive medical treatment, including vigorous efforts to support organ function and vital signs. In the words of a position paper by the American Society of Critical Care Medicine, "*the ICU serves as a place for monitoring and care of patients with potentially severe physiological instability requiring technical and/or artificial life support*”[1]. According to the Israel Medical Association, minimal requirements for an ICU include a manager, a physician for every six beds, a nurse for every two beds, availability of consultants (such as nephrology or infectious diseases), availability of specific medical equipment (such as mechanical ventilators), and auxiliary services such as laboratory testing and medical imaging [2]. There are different sorts of Intensive Medical Treatments (IMTs) that may be provided; among the most common are the administration of vasopressors (intravenous medications intended to support blood pressure and adequate circulation), mechanical ventilation, and the use of Bi-level Positive Airway Pressure (BiPAP) as an alternative to mechanical ventilation.

While these interventions would ideally be provided in an ICU, ICU beds are in short supply and demand often exceeds supply. In Israel, the shortage is even more severe compared to other developed countries. According to Israel’s Ministry of Health 2021 data for general hospitals, out of 16,180 licensed hospital beds, 758 (4.7%) were intensive care beds [3]. According to the OECD, as of 2020, Israel had 2.07 Acute Care beds (a broader term than ICU beds) per 1,000 people compared to an average rate of 3.55 such beds across 24 other OECD countries, and was ranked fourth-to-last on this metric [4].

Due to a pervasive shortage of ICU beds in Israel as well as in many other countries, some Intensive Medical Treatments (IMTs) may be provided in other settings. These settings may go by different names, such as Intermediate-Care Units, high-dependency care units, or step-down units. These settings have been defined as providing “*an intermediate level of clinical care between a general ward and intensive care*", and will be referred to in this paper as an Intermediate-Care Unit [5–9]. These units are intended for patients whose condition may exceed the abilities of a General Bed, but is not complex enough to warrant an ICU bed. However, in some cases, patients are placed in the Intermediate-Care Unit instead of the ICU due to a lack of available ICU beds. Studying this issue is complicated by the fact that the definition of Intermediate-Care Unit is less well-developed and precise than the definition of an ICU, both in Israel and elsewhere.

Due to the perpetual and pervasive shortage of ICU beds, as well as the variable demand for them, admission to the ICU is made at the discretion of senior ICU physicians based on a myriad of considerations, including the patient's medical condition, treatment requirements, availability of beds, and concurrent requests. Therefore, patients in a similar situation, with a similar level of illness, may be more or less likely to be admitted to the ICU, depending on bed availability at any given time [1, 10–12]. However, even when the ICU is at capacity, some patients still have intensive medical requirements, thus necessitating the internal medicine wards to adapt and provide this care to the best of their abilities, whether in an Intermediate-Care Unit or even in a regular ward bed.

Using a population of patients admitted to internal medicine at a large, tertiary referral hospital, this study examined the setting in which patients received Intensive Medical Treatments (IMTs): mechanical ventilation, daytime bi-level positive airway pressure (BiPAP), or vasopressor therapy, according to bed type (i.e., ICU, Intermediate-Care Unit, or general-bed). This study is intended to provide an insight into issues of crowding and treatment choices on internal medicine services in Israel.

### Description of facilities at Shaare Zedek medical center

Shaare Zedek Medical Center (SZMC) is a tertiary care medical center containing, as of 2019, 806 inpatient beds. Of these, 281 beds are for internal medicine patients, 202 for surgical patients, and 139 for maternity patients [13]. SZMC serves the population of Jerusalem, as well as the surrounding area.

In 2019, the hospital had a total of 38 intensive care beds: 25 general intensive care beds, and the remainder cardiac intensive care beds [13]. The intensive care unit is under the responsibility of board-certified critical care doctors, and the nursing staff in the units is required to undergo an intensive care course. The decision of which patients to admit to the ICU is made by the intensive care doctors, and is based on clinical judgment. The goal of the admission decision is to provide ICU beds to those patients who will benefit most from hospitalization in the ICU.

Intermediate-Care Units, known in Hebrew as nitur, are part of, and staffed by, the internal departments. SZMC has four internal medicine departments. Three of these departments have a 5-bed Intermediate Care Unit. In addition, there is one acute geriatric ward with a 5-bed Intermediate-Care unit, for a total of 20 intermediate-care beds in the hospital. Patients in these Intermediate-Care Unit beds are taken care of by the physicians in the department, who are not critical care specialists, although all have gone through rotations in intensive care units as part of their training. The nursing staff in the Intermediate-Care Unit are department staff as well, and usually are critical care qualified and trained, or at least have seniority and experience in treating these patients, with nurse-to-patient ratio of 1:5. While a ratio of 1:3 or 1:4 may be more common elsewhere, a ratio of 1:5 is prevalent here due to a lack of resources. Respiratory therapists are involved in patient care, especially for patients with respiratory issues, but are physically present less often on the wards and in the Intermediate Care Unit than they are in the ICU.

The dataset for this study extends through December 31, 2019. Therefore, it is important to give a sense of what has changed at SZMC since the time of the study. Since 2019 and following the COVID-19 epidemics, 42 additional licensed beds were added to SZMC. Of these 42 beds, 17 were added to the internal medicine wards, and one ICU bed was added [14].

### Patients monitoring

Because this manuscript is about different levels of hospital care, it is important to give the reader a sense of what level of monitoring is available at each level at SZMC, as summarized in Table 1.

**Table 1:** Monitoring level available at the different beds at SZMC

	ICU	Intermediate unit		General-bed
*Vital signs monitoring*				
Personal alarm	✔	✔		✔
Central monitor system	✔	✔		
Central monitor alarm system	✔	✔		
*Ventilator/infusion pumps*				
Personal alarm	✔	✔		✔
Central monitoring system				
*Central monitoring alarm*				
Patient-nursing staff ratio	1:2	1:5		1:9–1:12
Direct line of sight to patient at all times	✔	✔		

Level of monitoring available at different levels of acuity for internal medicine patients at SZMC

In both the intermediate unit and the ICU, vital signs are monitored using personal monitors connected to a central station. Heart rate, pulse oximetry, and blood pressure are continuously measured for all patients. In addition, an EKG and capnometer can also be connected if necessary. Each patient station has a monitor with an alarm system, however, in the ICU, unlike the intermediate unit, the alarm system is also connected to the central station, thus enabling additional supervision.

In contrast, monitoring the vital signs of a patient in a general ward bed is performed by their nurse. Patients may have a bedside monitor for vital signs such as heart rate and pulse oximetry, and these devices may have audible alarms, but they are not connected to a central system. Vital signs are usually taken at a greater frequency in patients who are sicker, such as every two hours.

In all units, if mechanical ventilators and infusion pumps are needed, each patient is treated using a personal unit where the medical parameters are adjusted by the medical staff according to patient's needs. These units are not connected to a central station. Nurses are responsible for hearing the alarms that these devices (ventilators and infusion pumps) may emit; their alarms are not connected to a central system.

Both in the ICU and in the intermediate unit, a nurse is present throughout the day and is responsible for the ongoing care of the patients, and can call for reinforcement if necessary. On the general ward, nurses are not in a line of sight to the patient at all times, as is the case in the ICU and the intermediate care unit. The patient-nursing staff ratio in the ICU is 1:2. This ratio drops to 1:5 in the intermediate unit. In the general ward, this ratio is between 1:9 and 1:12, with every effort made to assign extra effort to sicker patients as staffing allows.

## Methods

### Database

In this retrospective observational cohort study, we used data from SZMC, a large tertiary referral hospital in Jerusalem that serves a varied population in terms of ethnicity and socioeconomic status. The study included 56,002 hospitalizations between 01.01.2016 – 31.12.2019. These dates were chosen to allow us to study care under usual conditions, prior to the influence that COVID-19 had on the Israeli medical system. The study unit was hospitalizations rather than patients since some patients were admitted more than once.

For the purposes of this study, “internal medicine wards” were defined as the four formal internal medicine departments (A, B, C, and D), geriatrics, cardiology, and the observation/short stay unit of the emergency room. The rationale for including these additional units is that some Israeli hospitals do not have such units, and therefore the patients hospitalized at SZMC in these wards would be part of the population served by internal medicine in a different hospital setting. In order to capture the entire spectrum of internal medicine patients, they are included here. This study was approved by the research ethics committee of SZMC. (Approval number—SZ-0361-21(

### Variables

Patients’ deidentified medical information was extracted from the hospital's electronic medical record and included: dates of hospitalization and discharge, date of birth, sex, all wards the patient visited during the hospital stay, IMTs received (details below), and relevant outcomes such as in-hospital mortality and length of stay. We characterized all admissions as elective or emergency admissions. Because elective admissions directly to internal medicine at SZMC are extremely rare, essentially all of the patients with elective admissions were originally admitted for a surgical procedure, and eventually were transferred to the internal medicine service due to other issues that arose during their hospital stay. To characterize patients’ level of comorbid illness, we identified Elixhauser diagnosis groups, using the lists of ICD-9 codes as per the original paper by Elixhauser and colleagues [15].

We identified three IMTs, received at any time during the hospitalization, which formed the focus of this study: (a) receipt of any vasopressor (b) receipt of mechanical ventilation, and (c) receipt of bi-level positive airway pressure (BiPAP) between 8 AM and 8 PM. Vasopressors were given via volumetric infusion pumps, at any time during the hospitalization, and included adrenaline, dobutamine, dopamine, milrinone, noradrenaline, phenylephrine, and vasopressin. The reason to focus on daytime BiPAP is that BiPAP received at night may be for disordered breathing during sleep (i.e., obstructive sleep apnea), but BiPAP received during the day is presumably intended as a substitute for invasive mechanical ventilation.

We also characterized patients based on the type of bed in which they were hospitalized – general-bed only, Intermediate-Care Unit, ICU, or Both (Intermediate-Care and ICU, in either order).

## Analyses

We examined which proportion of patients in each group (general-bed, Intermediate-Care, ICU, Both) received which of the IMTs (none, one, different combinations of two of them, or all three). We compared means for continuous variables (age and length of stay) using one-way ANOVA tests and proportions for categorical variables (sex, elective admission, in-hospital mortality) using chi square tests. Analyses were performed using SPSS version 24 and RStudio version 1.3.1093.

## Results

A total of 56,002 hospitalizations were documented, with some patients hospitalized more than once. Of these hospitalizations, 1627 (2.9%) spent at least some time in the Intermediate-Care Unit, 1,983 (3.5%) spent at least some time in the ICU, and 226 (0.4%) were treated both in the Intermediate-Care Unit and the ICU.

Table 2 shows the characteristics of the patients included in the data file, considering each hospitalization as a separate incident and therefore counting some patients more than once. All between group differences presented throughout the results section are statistically significant (*p* < 0.001). A majority of hospitalizations were male (53%). Men were overrepresented among ICU patients (64% were male). The average age of our sample was 70.9 (SD 17.8). Intermediate-Care Unit patients were older than this average (mean age 75.1, SD 16.0), and ICU patients younger (mean age 69.1, SD 15.3).

**Table 2:** Patients Characteristics

	Bed type					
Characteristic	Overall *n* = 56,002	General-Bed *n* = 52,166	Intermediate-Care unit *n* = 1,627	ICU *n* = 1,983	Both *n* = 226	*p*-value^1^
*Sex *(*n;* %)						< 0.001
Male	29,955 (53%)	27,723 (53%)	815 (50%)	1,279 (64%)	138 (61%)	
Female	26,047 (47%)	24,443 (47%)	812 (50%)	704 (36%)	88 (39%)	
Age (Mean; SD)	70.9 (17.8)	70.8 (17.9)	75.1 (16.0)	69.1 (15.3)	67.1 (14.5)	< 0.001
*Hospitals Length of Stay (Days)*						
(Median; IQR range)	4 (2, 7)	3 (1, 6)	12 (2, 22)	9 (2, 16)	27 (11, 44)	< 0.001
Unit LOS (Mean Days)	–	–	5.5	4.2	–	
In-Hospital Mortality (n; %)	3,865 (6.9%)	3,199 (6.1%)	365 (22%)	231 (12%)	70 (31%)	< 0.001
Elective Admission (n; %)	7,899 (14%)	7,161 (14%)	14 (0.9%)	718 (36%)	6 (2.7%)	< 0.001
*Num elixhauser comorbidities (n; %)*						< 0.001
0	13,491 (24%)	12,929 (25%)	112 (6.9%)	440 (22%)	10 (4.4%)	
1	11,168 (20%)	10,556 (20%)	244 (15%)	336 (17%)	32 (14%)	
2–4	25,157 (45%)	23,180 (44%)	941 (58%)	908 (46%)	128 (57%)	
5 +	6,168 (11%)	5,501 (11%)	330 (20%)	299 (15%)	56 (25%)	

Characteristics for 56,002 internal medicine admissions to Shaare Zedek Medical Center, Israel (2016–2019). Hospitalizations are divided into those who were treated in a General-Bed, the Intermediate Care unit, Intensive Care Unit (ICU) or Both (Intermediate-Care or ICU). Cell count (%) or Median (IQR) for LOS, and mean (SD) for age are given

The level of comorbidity also varied by bed location as measured by the number of Elixhauser Comorbidity Diagnosis Groups (Table 2). Patients hospitalized in both the ICU and the Intermediate Care Unit were the most likely to have two or more Elixhauser comorbid conditions (82%), followed by Intermediate-Care Unit patients (78%). Only 62% of patients hospitalized in the ICU had two or more Elixhauser comorbid conditions.

Hospital total length of stay (LOS) also differed markedly based on bed location. The overall median LOS for the sample was 4 days (IQR 2,7). ICU patients had a longer LOS (median 9, IQR 2,16), and Intermediate-Care Unit patients had an even longer LOS (median 12 days, IQR 2,22). Patients who spent time in both the ICU and Intermediate-Care Unit had the longest LOS (median 27 days, IQR 11,44).

Table 3 presents the percentage of patients receiving the various IMTs (vasopressors, mechanical ventilation, or daytime BiPAP) based on their bed location. Among general-bed patients, the rate of receiving any of these interventions was relatively low, although not negligible (7%). ICU patients received these interventions at a higher rate (37% received at least one of them), but Intermediate-Care Unit patients received them at an even higher rate than that (52% received at least one). Furthermore, while 12% of ICU patients received dual intensive interventions, and 2.5% received all three, in the Intermediate-Care Unit these rates were even higher (16.0% and 6.9%, respectively).

**Table 3:** Hospitalizaion bed and intensive medical treatments distribution table

Intensive medical treatments (IMTs)		Bed type				Total
		General-bed	Intermediate-care unit	ICU	Both	
None (*n*)		48,506	780	1245	55	50,586
Row Percentage:* (Bed type share receiving Intensive-Treatment)		95.9%	1.5%	2.5%	0.1%	**100%**
Column Percentage:† (Intensive-Treatments share received by bed type)		93%	47.9%	62.8%	24.3%	90.3%
BiPAP (*n*)		1552	157	57	10	1776
Row Percentage: *(Percentage of this treatment received in this bed type)		87.4%	8.8%	3.2%	0.6%	**100%**
Column Percentage: † (percentage of patients in this bed type receiving this treatment)		3%	9.6%	2.9%	4.4%	3.2%
Ventilation (*n*)		688	161	283	30	1162
Row Percentage: *(Percentage of this treatment received in this bed type)		59.2%	13.9%	24.4%	2.6%	**100%**
Column Percentage: † (percentage of patients in this bed type receiving this treatment)		1.3%	9.9%	14.3%	13.3%	2.1%
Vasopressors (*n*)		827	158	109	16	1110
Row Percentage: *(Percentage of this treatment received in this bed type)		74.5%	14.2%	9.8%	1.4%	**100%**
Column Percentage: † (percentage of patients in this bed type receiving this treatment)		1.6%	9.7%	5.5%	7.1%	2%
Bipap & ventilation (*n*)		55	66	33	9	163
Row Percentage: *(Percentage of this treatment received in this bed type)		33.7%	40.5%	20.2%	5.5%	**100%**
Column Percentage: † (percentage of patients in this bed type receiving this treatment)		0.1%	4.1%	1.7%	4%	0.3%
Bipap & vasopressors (*N*)		136	42	21	5	204
Row Percentage: *(Percentage of this treatment received in this bed type)		66.7%	20.6%	10.3%	2.5%	**100%**
Column Percentage: † (percentage of patients in this bed type receiving this treatment)		0.3%	2.6%	1.1%	2.2%	0.4%
Ventilation & vasopressors (n)		328	151	186	50	715
Row Percentage: *(Percentage of this treatment received in this bed type)		45.9%	21.1%	26%	7%	**100%**
Column Percentage: † (percentage of patients in this bed type receiving this treatment)		0.6%	9.3%	9.4%	22.1%	1.3%
All three therapies (*n*)		74	112	49	51	286
Row Percentage: *(Percentage of this treatment received in this bed type)		25.9%	39.2%	17.1%	17.8%	**100%**
Column Percentage: † (percentage of patients in this bed type receiving this treatment)		0.1%	6.9%	2.5%	22.6%	0.5%
Total (*n*)		52,166	1627	1983	226	56,002
Row Percentage: *(Percentage of this treatment received in this bed type)		93.2%	2.9%	3.5%	0.4%	**100%**
Column Percentage: † (percentage of patients in this bed type receiving this treatment)		**100%**	**100%**	**100%**	**100%**	**100%*%***

The bolding and underlining is emphasize that the rows add up to 100%

Chi-square for entire table: *p* < 0.001

^*^ Row precent—% of hospitalizations in this bed type that received this specific IMT

^†^ Column precent—% of hospitalizations that received this specific IMT that were in this bed type

Proportion of hospitalizations receiving Intensive Medical Treatments by bed type for 56,002 internal medicine admissions to Shaare Zedek Medical Center, Israel (2016–2019)

**Fig. 1:**
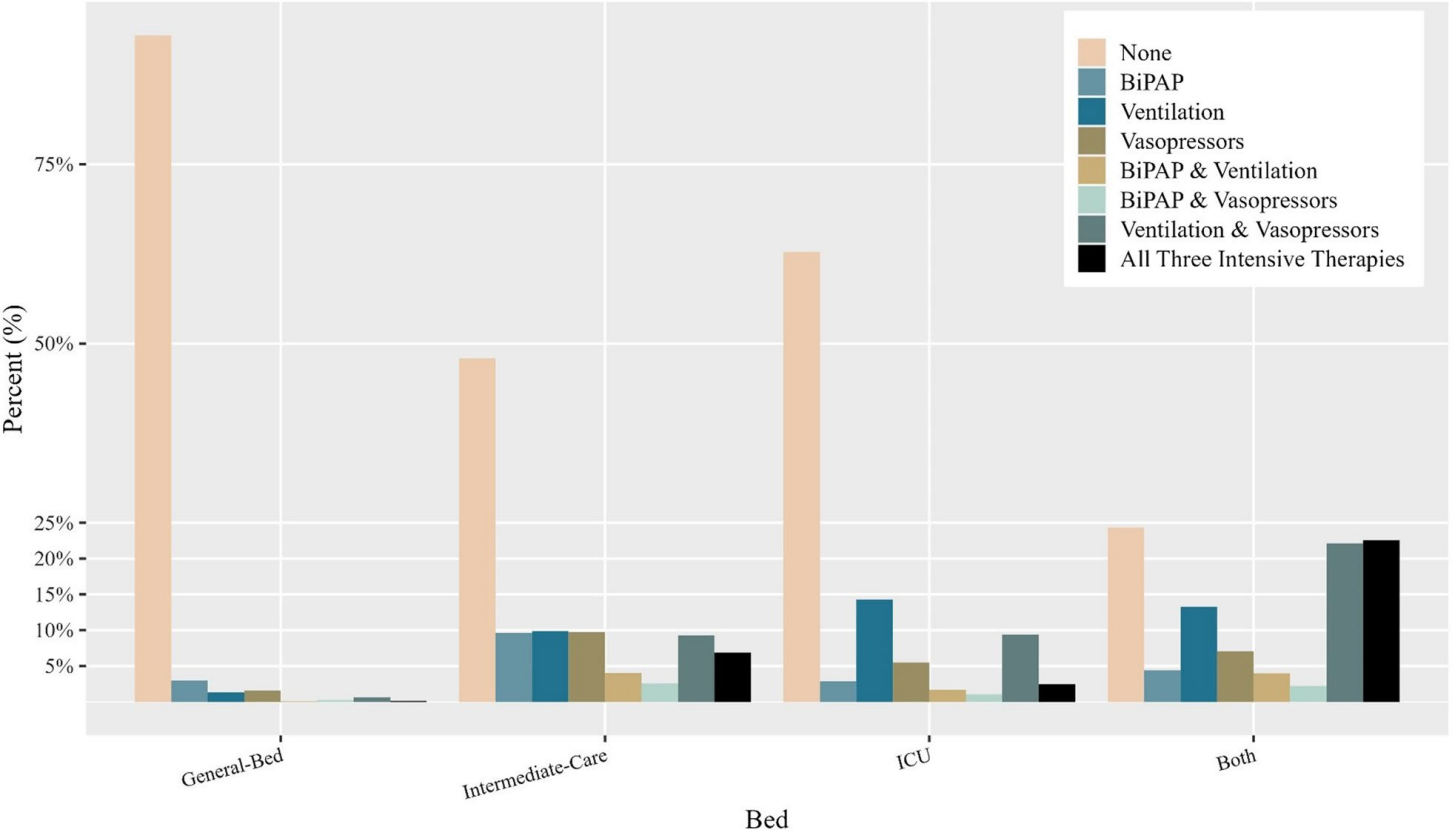
Intensive Medical Treatment distrubution proportion by bed type (%). For 56,002 internal medicine admissions to Shaare Zedek Medical Center, Israel (2016–2019*)*

This is further demonstrated in Fig. 1, which presents the relative share of each IMT by hospitalization bed. In the figure, one can see that most patients did not receive any IMTs, even in more intensive settings, with the exception of the small group of patients treated in both Intermediate-Care and ICU. In Intermediate-Care Unit, the IMTs most commonly received were single treatments (9.6–9.9%), dual treatment with ventilation and vasopressors (9.3%), followed by treatment with all three IMTs (6.9%). In contrast, among ICU patients, the most common IMTs were ventilation, followed by ventilation and vasopressors, and vasopressors alone (14.3%, 9.4% and 5.5%, respectively).

**Fig. 2:**
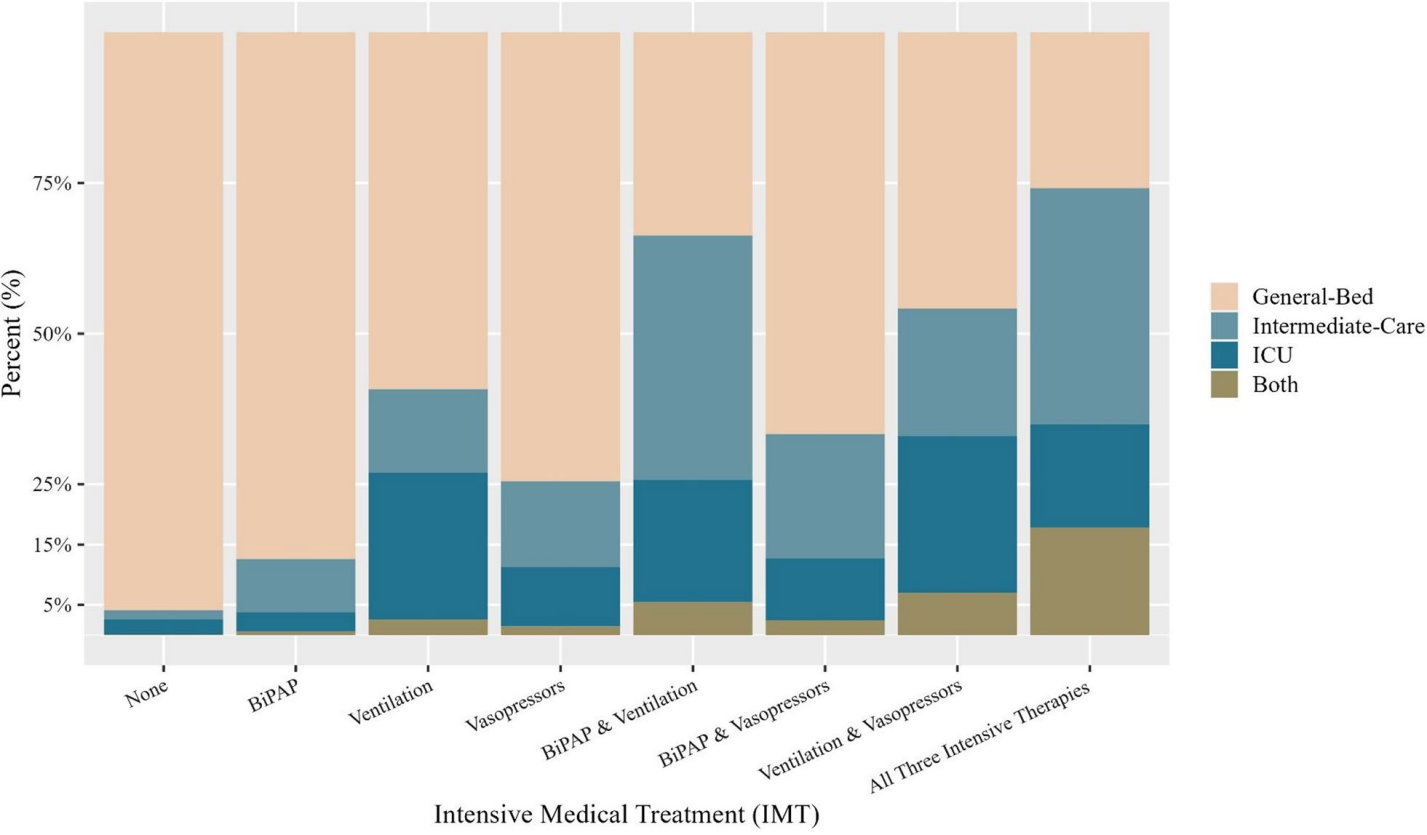
Proprtion of patients in different bed types receiving Intensive Medical Treatment. For 56,002 internal medicine admissions to Shaare Zedek Medical Center, Israel (2016–2019)

Figure 2 shows the proportion of treatments by bed location. While ICU, Intermediate-Care Unit, and ICU/Intermediate Care patients were indeed overrepresented among those receiving IMTs, the majority of patients receiving most IMTs received them in a general-bed. For example, 87.4% of patients receiving daytime BiPAP, 74.5% of patients receiving vasopressors, and 66.7% of patients receiving both BiPAP and vasopressors received them in a general-bed setting. Surprisingly, the second highest absolute number of patients receiving each IMT (after general-bed) were Intermediate-Care unit patients, with ICU patients coming in third.

We examined whether there was a difference in where patients received IMT at different times of year. We found that the location where patients received IMT differed between the summer and the winter months (*p* = 0.008, see Table 4). Throughout the year, the percentage of IMT given in a general bed never fell below 64%. However, in the winter half of the year (November–April), this proportion ranged from 67 to 72%, compared to 64–66% in the summer months. In contrast, the proportion of IMT received in the ICU and the Intermediate Care Unit were higher in the summer months. The proportion of IMT given in the ICU ranged from 13 to 16% in the summer, compared to 11–14% in the winter. Similarly, the proportion of IMT given in the Intermediate Care Unit ranged from 15 to 18% in the summer, compared to 13–16% in the winter.

**Table 4:** Number of patients receiving intensive medical treatment by bi-month and bed type

	Jan–Feb^1, 2^	Mar–Apr^1, 2^	May–Jun^1, 2^	Jul–Aug^1, 2^	Sep–Oct^1, 2^	Nov–Dec^1, 2^
*Bed*						
Total patients receiving IMT (% of total patients)	*N* = 954 (9.8%)	*N* = 931 (10%)	*N* = 806 (8.6%)	*N* = 809 (8.9%)	*N* = 798 (9.2%)	*N* = 1,118 (11.4%)
General-bed	684 (72%)	644 (69%)	530 (66%)	521 (64%)	530 (66%)	751 (67%)
Intermediate-care	127 (13%)	142 (15%)	124 (15%)	127 (16%)	147 (18%)	180 (16%)
ICU	105 (11%)	123 (13%)	124 (15%)	129 (16%)	105 (13%)	152 (14%)
Both	38 (4.0%)	22 (2.4%)	28 (3.5%)	32 (4.0%)	16 (2.0%)	35 (3.1%)

^1^ *n* (% of patients receiving IMT)

^2^ *p*.value = 0.008; Pearson's Chi-squared test

Number of patients receiving Intensive Medical Treatments by month and bed type for 56,002 internal medicine admissions to Shaare Zedek Medical Center, Israel (2016–2019)

## Discussion

In this retrospective descriptive study, we used data from 56,002 internal medicine hospitalizations over four years at a large tertiary care hospital in Israel to examine the distribution of intensive medical therapies by bed location. The majority of patients in most settings did not receive any IMTs. While patients in advanced-care settings received IMT at a higher rate than those on the general ward, in an absolute sense, most IMTs were provided in a general-bed setting. The exception to this was with BiPAP-ventilation and treatment with all three IMTs, which were delivered most often in Intermediate-Care Unit, followed by general-bed and the ICU. These findings are contrary to our expectations that most IMTs would be delivered in an ICU, followed by Intermediate-Care Unit. One possible explanation is that ICU beds were reserved for those most likely to benefit from treatment in such a bed, therefore the more complex patients were treated in a general-bed or an intermediate-care bed. This can also explain the higher mortality rates in the Intermediate-Care Unit compared to the ICU (22% vs 12% respectively).

Some previous studies have assessed the role of an Intermediate-Care Unit in providing intensive care, or have compared Intermediate-Care Unit and ICU, examining factors such as hospitalization outcomes, mortality rates, and costs. In addition, most previous studies have focused on the care of a specific condition, or on the delivery of a specific medical treatment [6, 16–20]. However, to the best of our knowledge, no previous study has tabulated the proportion of patients in each setting who received various IMTs, as well as the proportion of patients receiving each IMT who received it in this or that setting.

Our study indicates that most of the patients who received IMT actually received them in a general-bed, rather than a specialized unit. Our findings correspond with other work from another hospital in Jerusalem, which examined trends in mechanically ventilated patients over 20 years and found an increase from 31 to 67 in the number of ventilated patients per day. In that study, the authors attributed the majority of the increase to more patients being ventilated in the internal medicine wards (which increased from 4 to 24 ventilated patients per day) [21]. Such a change is not without consequences, because patients in need for respiratory support that receive it in the internal ward rather than the ICU show worse hospitalization outcomes, and higher mortality rates [22–25]. For example, a study from our institution showed a 74% in-hospital mortality rate for patients mechanically ventilated who were rejected from the ICU and were treated instead on the internal medicine wards [26]. However, such a study is also likely to be explained in part by confounding by severity, and so study designs that can support causal inference should be used in future studies.

Unit beds—both Intermediate-Care and especially ICU beds—are a scarce resource; therefore, considerable attention should be devoted to using them wisely. More rational use can help reduce the chance that patients in need of escalations of care may need to remain in a lower-intensity setting solely because all the higher-intensity beds are currently occupied. This, in turn, could help contribute to better patient outcomes and probably lower cost of care. However, even the wisest stewardship of resources cannot fully compensate for severe under-supply of high-intensity beds beyond a certain point. Our finding that there was a difference between winter and summer months strongly supports the supposition that there are simply too few high-intensity beds at SZMC. In the winter months, due to an increase in seasonal respiratory viruses, there is even greater strain on hospital resources. We saw this reflected in the absolute number of patients receiving IMT treatments, which is higher by 100–300 patients in the winter compared to the summer months. This phenomenon was also reflected in the fact that a somewhat greater percentage of IMT were received in a general-bed in the winter than in the summer months. However, even during the summer, the great majority of IMT were received in a general-bed. This points to a severe and persistent undersupply of high-intensity beds, and not a mild undersupply that only manifests during the winter.

One of the factors driving our results may be the issue of exactly what level of services are provided by an Intermediate-Care Unit. There is great variation between different hospitals and countries in the definition of what constitutes an Intermediate-Care Unit. In a systematic review that combined studies regarding 39 Intermediate-Care Units in 11 countries, many differences were found between the various Intermediate-Care Units; for example, unit names, number of beds, nurse-to-patient ratio, medical staffing, management, location in the hospital, and the medical specialties in charge of staffing the Intermediate-Care Unit [27]. The study concluded with the statement that “*supportive modalities differ between [*Intermediate-Care Units]*, although all offer continuous monitoring and respiratory support, while mechanical ventilation and the use of multiple vasoactive medications [may not be provided in some* Intermediate-Care Units*]*”.

It is worth noting that the nurse-patient ratio at SZMC is 1:2 for the ICU, 1:5 for the Intermediate-Care Unit, and ranges from 1:9 to 1:12 in the general-bed depending on the shift and manpower availability. Thus, while IMT may be provided in all three settings, these differences in nurse-patient ratio must surely have an impact on how closely IMT can be monitored and managed. The results of this study, combined with the continual shortage of ICU beds, which is not unique to our setting, challenge the usual vision of intensive care as the primary location where IMTs are provided. If anything, this presents an opportunity for us to re-examine where and how we provide IMTs.

IMT requires special equipment, staff, and appropriate conditions, such as special units dedicated to delivering these treatments. The highest level of care is the ICU. ICU resources are monitored to some extent by the Ministry of Health as well as the medical centers themselves. In contrast, the delivery of IMT in Intermediate-Care Unit and general-bed is supervised by the medical center itself, and may vary quite a bit. At the time of our study period, 4.7% of SZMC general hospital beds were intensive care beds, similar to other Israeli hospitals with approximately the same number of beds (i.e. 600–1,000 beds); the other hospitals ranged from 4.2 to 6.4% [13]. Since 2019, Israel’s Ministry of Health has licensed 114 more intensive care beds across the country; however, despite this increase, the rate of intensive care beds in these hospitals with similar size did not change markedly. At the end of 2022, the proportion of ICU beds at those Israeli hospitals ranged from 3.6 to 7.2% [14], mostly unchanged from 2019. These numbers, showing that the situation at SZMC is comparable to other Israeli hospitals, suggest that the problems we documented here are pervasive, and not unique to SZMC.

Our results suggest patterns of care that may have evolved by accident in response to existing conditions, rather than the results of a deliberate policy regarding which care to provide where, and to whom. We believe that our study should prompt a deeper examination of patterns of intensive interventions, including the settings where they are delivered. It would certainly be worth directly making sure that the patterns we saw at SZMC are similar at other Israeli medical centers, as we think is likely the case. Afterwards, we believe there should be a national conversation about the resources currently available for delivering IMT, the current need for them across different institutions, and how policy can be developed and implemented to best match these in real time. It may be that Israel is so under-resourced in terms of ICU and Intermediate-Care Unit beds that an increase in beds would have to precede any effective effort to make such triage decisions more rational and explicit.

The main limitation of this study is that we cannot look into and comment on the clinical reasoning that went into each decision about where and how to treat each patient, since aggregated data was analyzed, without reviewing charts. Therefore, our study design does not allow us to examine whether the patients we studied would have done better or worse in a different care setting or level of care. In addition, we did not check what the occupancy was at any given moment in the various units, which could have affected the considerations of where to treat each patient. Finally, our study was limited to a single hospital. SZMC is a large hospital that treats a diverse set of patients, and is likely to be representative of many Israeli hospitals. However, critical care delivery patterns and Intermediate-Care Units may vary greatly between hospitals, and therefore, examining similar patterns of care at other hospitals, both in Israel and beyond, would certainly be a fitting topic for further study.

Nevertheless, despite these limitations, our results are eye-opening, and may suggest a need to add additional ICU and Intermediate-Care beds. In our study, the majority of patients who received some forms of IMT did so in a general-bed. This was particularly true for vasopressors, which were most often given in a general-bed. While we repeat that we did not review the charts, this certainly raises the possibility that some of these patients could have benefited from a more advanced care setting. We also found that patients in the SZMC Intermediate-Care Unit had longer lengths of stay and higher in-hospital mortality than patients in the ICU. This may imply that these patients had a higher level of acuity and worse prognoses, which in some cases may have caused them to be refused admission to the ICU. In turn, this may imply that futile interventions were provided on the wards for at least some patients who had not been accepted to the ICU. Further study is needed to better understand the clinical course of such patients. In a follow-up study, we intend to generate and test extended risk-adjustment models for these patients, focusing primarily on predicting outcomes of hospitalization including in-hospital mortality, LOS, need for IMT, and 30-day readmission. The present study was undertaken in part to examine the different components of our IMT outcome.

### Health policy implications

The present study raises several clear implications for health policy. First, our results call for an immediate examination of similar data across all of Israel’s hospitals, to confirm that the situation is similar at other facilities, as we think will be the case. Second, we recommend a comprehensive definition, at the national level, for what constitutes an Intermediate-Care Unit, how it should be defined, and how it should be staffed. This would include explicit admission criteria, number of beds, staff to patient ratios, minimal services to be offered, and how to measure quality and safety of care, all similar to the recommendations of the German Interdisciplinary Association for Intensive Care and Emergency Medicine [28]. Third, we recommend that if there is a need to add ICU and Intermediate-Care Unit beds, that Israel undertake to do so expeditiously and without delay. Finally, we recommend an examination of how advanced-care beds are currently allocated, as well as a plan to optimize and rationalize how they will be allocated going forward, to maximize patient benefit. However, as we said earlier, if the undersupply of advanced-care beds is severe enough, it may not be possible to design or implement a rational plan to determine who should receive this scarce resource.

## Conclusions

In conclusion, we examined the bed type where internal medicine patients received intensive medical interventions at a large tertiary-care hospital in Israel. We found that many intensive medical interventions were received in an unmonitored general ward bed. We also found that patients in an intermediate care unit setting had longer LOS and had poorer outcomes than patients in the intensive care unit, and in many cases were more likely to receive most intensive interventions. Our results should prompt a deeper examination of how treatments should be given to patients whose condition is deteriorating, and in which settings.

## Data Availability

We will share our statistical code and study protocol upon request. SZMC data are available upon reasonable request and after completing a data use agreement.

## Abbreviations

ICU: Intensive care unit
IMT: Intensive medical treatments
BiPAP: Bi-level positive airway pressure
OECD: The organisation for economic co-operation and development
SZMC: Shaare Zedek medical center
LOS: Length of stay
